# Organic contaminants as an ecological tool to explore niche partitioning: a case study using three pelagic shark species

**DOI:** 10.1038/s41598-019-48521-6

**Published:** 2019-08-19

**Authors:** Kady Lyons, Dovi Kacev, Antonella Preti, David Gillett, Heidi Dewar

**Affiliations:** 1 Georgia Aquarium, 225 Baker Street NW, 30313 Atlanta, Georgia; 2 Southwest Fisheries Science Center, La Jolla, California USA; 3 Southern California Coastal Water Research Project, Costa Mesa, California USA; 4 University of California Santa Cruz, Santa Cruz, California USA

**Keywords:** Community ecology, Ecophysiology

## Abstract

Chemical contaminant profiles are linked to an animal’s niche, providing a potential tool by which to assess resource partitioning in pelagic species. As proof of concept, we examined contaminant signatures in three species of sharks (*Isurus oxyrinchus*, *Prionace glauca*, and *Alopias vulpinus*) known to overlap in both space and time. Since these sharks comprise a predatory guild within the Southern California Bight (SCB), we predicted that species may partition spatial and dietary resources to limit the extent of competitive exclusion. Indeed, species were distinguishable by both total contaminant loads and their contaminant fingerprint, as random forest analysis found that species could be correctly classified 96% of the time. Our results demonstrate the utility of chemical analyses for ecological studies, and how contaminant tracers can be used in combination with traditional methods to elucidate how species may undergo niche partitioning to reduce competition for overlapping resources within predatory guilds.

## Introduction

Interspecific resource competition is one of the primary biotic drivers modifying species’ fundamental niches towards their realized niches within a community^1–3^. Classical ecological theory suggests that niche space must be divided among sympatric organisms in order for species to coexist without competitive exclusion^4–6^. These divisions can result in species utilizing different spatial or trophic resources, with the degree of overlap inversely proportional to the intensity of their competition for resources^7,8^. Sympatric predators often appear to share niche space; however, studies demonstrate that resource partitioning does occur within predator guilds^9,10^. This resource partitioning allows for a more biodiverse predator assemblage, which may help buffer ecosystems from major changes caused by top-down forcing in the case of individual species loss due to environmental fluctuation^11^.

Since ecological niches are complex, empirically determining niche partitioning in natural systems is nontrivial. Traditionally, niche partitioning is evaluated using tagging and/or gut content studies, where separation of species’ trophic or spatial resources can be identified through their movements^12,13^ or prey composition^14–16^. These methods are, however, logistically challenging in marine environments where organisms spend the majority of their time underwater and can be difficult to observe (tagging) or are dependent on capture directly after a meal (gut contents). To overcome these challenges, other tools are being used in conjunction with traditional methods. Chemical analyses, such as stable isotope analysis (SIA), are widely used to indirectly determine trophic and spatial resource partitioning^10,17^. However, depending on the tissues sampled, SIA can only give a snapshot of weeks to months, a similar temporal limitation of other ecological tools.

As the use of “non-traditional methods” is becoming more common place in ecological studies, toxicological analyses offer an alternative and complementary perspective for understanding how animals interact with their environments^18–22^. The release and spread of anthropogenic contaminants worldwide, in particular legacy organic contaminants such as polychlorinated biphenyls (PCBs) and pesticides, coupled with their high resistance to degradation, has resulted in contamination of even the most pristine environments^23,24^. Thus, these legacy hydrophobic organic contaminants represent reliably detectable chemical markers, particularly for long-lived predatory species that have a high propensity to accumulate these contaminants^25–27^. Similar to stable isotopes, organic contaminants are acquired mainly through diet and are incorporated into tissues based on each contaminant’s physio-chemical properties^28^. In addition, organic contaminants can offer some degree of spatial resolution as different areas have unique chemical signatures based on the history of direct contaminant release or atmospheric deposition^29^. These contaminant signatures are then incorporated into local biota, which will generally reflect the environment, assuming a majority of contaminants from the diet are absorbed and metabolism by the predator is limited so as to not alter these contaminant signatures. For instance, organisms utilizing the Southern California Bight (SCB) have a strong signature of a dichlorodiphenyltrichloroethane (DDT) metabolite (e.g. 4,4′-DDE) due to the high degree of DDT release into the marine environment in the 1900s^30^. Since species’ contaminant loads vary based on their use of spatial and trophic resources, chemical analysis is a potential tool to examine resource partitioning in sympatric species while taking aspects of their physiology into consideration^22^.

In the California Current Large Marine Ecosystem (CCMLE), *Isurus oxyrinchus* (Shortfin Mako), *Alopias vulpinus* (Common Thresher Shark), and *Prionace glauca* (Blue Shark) comprise a sympatric predator guild^12,31^, making them excellent candidates to determine whether toxicological tools can aid in our understanding of resource partitioning. Within the CCMLE, the SCB represents an important nursery area for these sharks, as juveniles of all three species are relatively abundant in the region^31,32^. Previous tagging and dietary analyses indicate that some degree of niche partitioning occurs among these three species. Spatially, *A*. *vulpinus* are the most coastal^32^, whereas *P*. *glauca* and *I*. *oxyrinchus* make more regular movements offshore^12^. With respect to diet, all three species demonstrate some degree of prey overlap, but species can be distinguished based on stomach content composition^16^. As such, we would predict these differences to be reflected in their contaminant signatures. In an accompanying study^33^, we previously demonstrate how aspects of species’ physiology and ecology can result in different lifetime contaminant accumulation trajectories by measuring a suite of legacy contaminants in liver tissue across a range of animal sizes. *P*. *glauca*, the species with the lowest estimated metabolic rate and least time spent nearshore, demonstrated low potential for lifetime accumulation, although adult sampling was limited. While *A*. *vulpinus* and *I*. *oxyrinchus* both demonstrated patterns of growth dilution during the juvenile stage, only *I*. *oxyrinchus* exhibited significant increases in liver contaminant concentration in adults, likely due to their high metabolic rate and relatively high trophic position. Species characteristics that may influence contaminant input (i.e. where animals feed, what they feed on or how often they feed) may also have the potential to influence species’ unique contaminant signatures. Therefore, we aimed to demonstrate the applicability of organic contaminants as a tool to examine the degree of niche partitioning among three species of sympatric sharks within the same predatory guild using contaminant information collected previously^33^.

## Methods

### Sample collection

Liver samples were opportunistically obtained from frozen samples archived at the Southwest Fisheries Science Center’s (SWFSC) from the juvenile Shortfin Mako/Blue Shark survey^31^ and Common Thresher Shark survey, the National Marine Fisheries Service (NMFS) West Coast Region Fishery Observer Program, participating drift gillnet fishermen, and recreational fishermen. A subset of these archived samples was then analyzed for organic contaminants (see below) in individuals selected to represent a range of sizes and sexes for *I*. *oxyrinchus*, *P*. *glauca*, and *A*. *vulpinus* sampled from the years 2011–2013. Archived samples were also supplemented with contaminant data from previously published reports^34–36^ from animals sampled in the same time frame (*i*.*e*., 2011–2013).

### Organochlorine contaminant analysis

Organic contaminants were analyzed at California State University Long Beach’s IIRMES facility following previously published methods^34^. Liver subsamples (0.5–1.0 g wet weight) from each animal were extracted for 14–16 hrs via a Soxhlet apparatus in 100% methylene chloride solution, followed by subsequent evaporation and sample purification by elution through an Alumina-B/Silica gel. Extracts were spiked with internal standards (4,4′-Dibromobiphenyl and 2,2′,5,5′-Tetrabromobiphenyl, Accustandard, Inc, New Haven, USA) and injected onto an Agilent gas chromatograph (GC; 6890N series) equipped with a mass selective detector (MSD; Agilent 5973 inert series, Santa Clara, USA). Ion peaks were then identified using gas chromatography mass spectrometry (GCMS) software for 54 PCB congeners (sum = tPCBs), DDT and its metabolites (4,4′-DDT, 4,4′-DDE, 4,4′-DDD, 2,4′-DDT, 2,4′-DDE, 2,4′-DDD; sum = tDDXs), and non-DDT chlorinated pesticides (24 compounds screened; sum = tPEST); total concentrations were expressed as the sum of all contaminant groups (tOCs). The limit of detection for all compounds was 1 ng/g. Part way through sample processing, the method at the laboratory where chemical analysis took place to include 4,4′-DDMU, a downstream break down product of 4,4′-DDE; thus, only a subset of samples (n = 44) included this metabolite and no samples from previous studies had this metabolite analyzed. Concentrations of this metabolite are reported for samples where it was measured, but were not included in overall analyses due to incongruency in measured congeners among samples. Lipid content was determined gravimetrically from split aliquots of the extracts; however, since neither wet nor lipid normalized concentrations were correlated with liver lipid content for any species (p ≥ 0.16) we reported values on a wet weight concentration basis.

To ensure quality control/quality assurance, all samples were spiked with recovery surrogate compounds (TCMX, PCB30, PCB112, PCB198) prior to extraction to measure extraction and recovery efficiency of procedures (recovery ranges for all four surrogates: 75 ± 10%). In addition, a certified reference material (Lake Michigan Trout tissue 1947, National Institute of Standards and Technology, Gaithersburg, USA), a pair of blank spikes (i.e. 400 ng of each measured congener), a blank (spiked only with recovery surrogates), and a liver sample replicate were run in tandem with each batch (i.e., ~every 24 samples). All compounds in the certified reference material were within ±30% of the reference value^37^. Recovery of measured compounds (n = 83) in blank spikes (n = 4) were 108 ± 7% for PCBs and 89 ± 20% for pesticides, with 4.2 ± 3.3% relative standard deviation between blank spike replicates. Blanks had high recovery of recovery surrogates (90 ± 28%) and showed little to no contamination during procedures (e.g. <2 ng/g of PCB153/PCB138); therefore, liver sample values were not blank-corrected. For liver samples where a replicate was run, the median relative significant difference between concentrations of quantifiable compounds within replicate pairs was 9.9 ± 2.8%, and mean concentrations were used for samples where replicates were available.

### Data analysis

Organic contaminants were compared among species in two ways to determine if species could be distinguished based on their contaminant concentrations or signatures. For our comparison of tOCs, individual contaminant concentrations were summed across compounds for each shark sample and expressed on a wet weight (ww) basis. tOCs were then compared among *I*. *oxyrinchus*, *P*. *glauca*, *and A*. *vulpinus* using a Kruskal-Wallis (KW) test followed by a Wilcoxon rank-sum test, with all sizes and sexes combined within species. Contribution of each contaminant group (*i*.*e*. tPCBs, tDDXs, and tPEST) was calculated as the proportion that each contaminant group contributed to the total contaminant load (tOCs). Differences in contaminant proportion was assessed using a regression modeling approach for dependent data with a beta distribution such as proportional data implemented in the *betareg* package in R (v 3.2.0).

To determine if species could be distinguished based on their contaminant signatures (*i*.*e*. relative differences in standardized contaminant proportion), measured contaminants for each individual were standardized by dividing each individual contaminant concentration by the sample’s own PCB153 concentration (a contaminant that is consistently detected in all species^38^) to obtain a ratio for each contaminant per individual. Since some contaminants had much larger concentrations than others (*e*.*g*. 4,4′-DDE), ratios were standardized via a z-score transformation by species. A classification random forest analysis was then used to determine if individual shark samples could be correctly assigned by species based on their z-score transformed contaminant profiles alone. Random forest analysis was conducted using the *randomForest* package in R. To determine species assignment, we set the number of trees (ntree) equal to 10,000, and to avoid biases caused by unequal sample sizes among the different species, we set the number of individuals used in the training set (sampsize) equal to eleven, which is half the number of individuals in the smallest group (*P*. *glauca*). To visualize the separation of species based on contaminant signatures we performed a non-metric multidimensional scaling (nMDS; *vegan* package in R) using contaminant ratios where each contaminant was standardized to the samples own PCB153 concentration and k was set to two.

## Results

**Figure 1:**
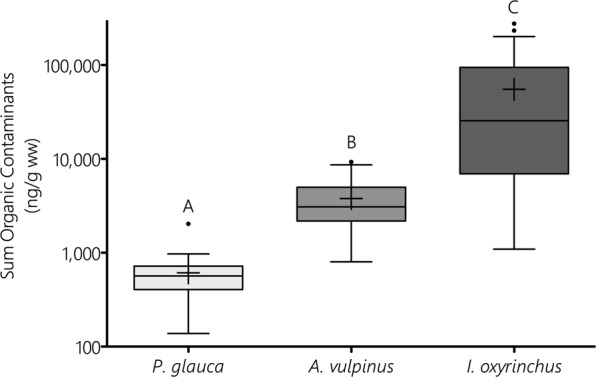
Comparison of sum tOCs measured in livers of *P*. *glauca*, *A*. *vulpinus*, and *I*. *oxyrinchus*. Different letters indicate significant differences, with an approximate 10-fold step-wise difference between species in terms of contaminant concentration. Crosses denote group means, and 25-50-75 quantiles are shown with whiskers and outliers plotted using the Tukey method.

Species showed significant differences in tOCs (KW, p < 0.001; Fig. 1) despite the inclusion of both sexes and a broad range of sizes, particularly for *I*. *oxyrinchus* and *A*. *vulpinus* that encompassed young-of-the-year through mature animals. *I*. *oxyrinchus* (n = 47, 53 to 337 cm fork length [FL]) had the highest tOCs (mean ± SD: 55,186 ± 66,968 ng^−1^ g ww), which were approximately 15 and 90 times greater than mean tOCs in *A*. *vulpinus* (n = 51, 63 to 283 cm FL, 3,781 ± 2,226 ng^−1^ g ww) and *P*. *glauca* (n = 22, 50 to 178 cm FL, 611 ± 382 ng^−1^ g ww), respectively. Besides having the lowest tOCs, *P*. *glauca* also had the fewest number of detectable contaminants measured (approximately 33 consistently measured contaminants out of a possible 84 screened), whereas *A*. *vulpinus* and *I*. *oxyrinchus* had much higher numbers (48 and 57, respectively).

While no information is available on the health of individuals sampled for this study, species risk to the potential negative effects of contaminant exposure may not be similar due to the magnitude differences in PCB concentrations across species. With respect to tPCB concentrations eliciting negative effects in mammals^39–42^, mean *I*. *oxyrinchus* consistently exceeded these thresholds regardless of whether wet or lipid normalized values were used, while mean *P*. *glauca* concentrations fell under these thresholds. Depending on the study, tPCBs concentrations in *A*. *vulpinus* either exceeded or fell below mammalian effect thresholds. Although studies are limited, environmental PCB exposure has the capability of eliciting negative physiological responses in elasmobranchs^43,44^. Compared to these studies, mean tPCBs in all three shark species exceed the lowest wet weight tPCB value in *U*. *halleri* and two of the three species exceed lipid normalized concentrations^43^. Considering the disparity in tPCB concentrations, it is likely that physiological risk to contaminant exposure is not equitable across these shark species.

For all species, tDDXs comprised the greatest proportion of the tOC. DDX contribution was highest in *A*. *vulpinus* (69.7 ± 2.8%) and was significantly greater than both *P*. *glauca* and *I*. *oxyrinchus* (Pseudo R-squared: 0.06283, z-value = 2.57, p = 0.009), which had similar DDX contributions to the total load (63.5 ± 3% and 63.2 ± 2.5%, respectively). While 4,4′-DDE contributed the most to tDDX concentrations (*Av* = 87 ± 8%; *Pg* = 90 ± 4%; *Io* = 96 ± 5%) and was the most frequently detected DDX (100% for each), species had differing patterns for the next most frequently detected DDXs. For example, *P*. *glauca* had the lowest frequency of detection for 2,4′-DDX compounds (21% of the time) compared to *A*. *vulpinus*, which had the highest (69%), with *I*. *oxyrinchus* intermediate (53%). For samples where 4,4′-DDMU was measured, *P*. *glauca* had the lowest frequency of detection (1/3) and concentrations (1.67 ng/g ww), whereas both *A*. *vulpinus* and *I*. *oxyrinchus* had high rates of detection (18/22 and 18/19, respectively) and comparable mean concentrations (42 ± 13 ng/g ww and 57 ± 85 ng/g ww), although concentrations varied by an order of magnitude among *I*. *oxyrinchus*.

PCB compounds were the next highest contributor to tOCs, and contributions were not significantly different among the three species. *I*. *oxyrinchus* had the most variability in PCB contribution (33 ± 12.6%), followed by *P*. *glauca* (25 ± 8.9%) then *A*. *vulpinus* (27 ± 7.8%). Among PCBs that contributed the most to total PCB loads, PCB153 and PCB138 consistently ranked first and second among species; however, the relative contribution of these congeners to tPCBs differed (Table 1). Of the top 10 contributing PCBs, species differed in both PCB congener rank order and relative contributions of these congeners to tPCB loads (Table 1). Detection frequencies also varied among species. *I*. *oxyrinchus* had the most congeners detected in one or more samples (91% of 54 congeners measured) as well as consistently higher detection frequencies of congeners across samples (71 ± 30% of samples), followed by *A*. *vulpinus* (% congeners detected = 87%; average detection frequency = 60 ± 32%) with the least number (83%) and lowest frequency of detections (36 ± 36%) in *P*. *glauca*.

**Table 1:** Mean ± standard deviation contributions to total PCB loads of the top ten most frequently detected PCB congeners for each species.

*P*. *glauca*			*I*. *oxyrinchus*			*A*. *vulpinus*		
Congener	Mean	StDev	Congener	Mean	StDev	Congener	Mean	StDev
PCB153 (1)	0.31	0.097	PCB153 (1)	0.25	0.022	PCB153 (1)	0.21	0.049
PCB138 (1)	0.22	0.052	PCB138 (1)	0.17	0.020	PCB138 (1)	0.15	0.031
PCB187 (1)	0.062	0.026	PCB180 (1)	0.086	0.018	PCB187 (1)	0.067	0.013
PCB180 (0.91)	0.083	0.032	PCB187 (1)	0.083	0.0092	PCB101 (1)	0.058	0.012
PCB170 (0.91)	0.038	0.030	PCB118 (1)	0.054	0.010	PCB99 (1)	0.038	0.0097
PCB118 (0.86)	0.096	0.024	PCB101 (1)	0.039	0.011	PCB110 (1)	0.033	0.0088
PCB183 (0.82)	0.022	0.015	PCB170 (1)	0.028	0.0082	PCB118 (0.98)	0.063	0.014
PCB99 (0.77)	0.067	0.0	PCB99 (1)	0.027	0.0095	PCB183 (0.96)	0.020	0.0072
PCB101 (0.77)	0.057	0.032	PCB183 (1)	0.026	0.0054	PCB158 (0.96)	0.011	0.015
PCB110 (0.5)	0.036	0.016	PCB201 (1)	0.019	0.0071	PCB180 (0.91)	0.072	0.022

For each shark sample, the contribution proportion of each detected PCB congener was calculated as PCBX/tPCBs and mean ± standard deviation was calculated across samples within species. Detection rates (within species) are shown in parentheses next to each congener. For *I*. *oxyrinchus*, two additional contaminants were detected in all samples (PCB110 and PCB158) that contributed 1.1 ± 0.5% and 0.85 ± 0.19% on average to total PCB burdens.

Of the non-DDT pesticides, trans-nonachlor consistently contributed the most to concentrations for all three species (*Io*: 54 ± 14%, *Av*:49 ± 15%, *Pg*: 40 ± 13%). However, for the next two chemicals contributing to non-DDT pesticide concentrations, species varied in both the type of contaminant and its proportion. For example, in *I*. *oxyrinchus* mirex (22 ± 12%) and cis-nonachlor (15 ± 5%) ranked second and third in proportional contribution, whereas cis-nonachlor (20 ± 7%) and alpha-BHC (19 ± 6%) were important to *A*. *vulpinus* pesticide concentrations and oxychlordane (23 ± 15%) and alpha-chlordane (20 ± 7%) to *P*. *glauca*.

**Figure 2:**
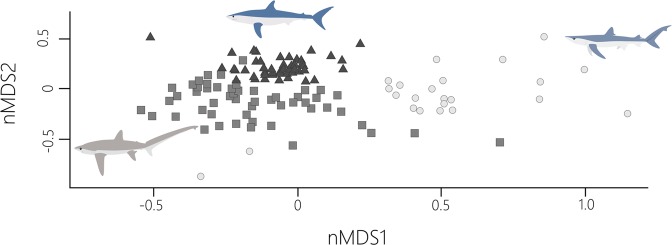
When contaminant signatures were compared along two dimensions, species showed clear separation from each other (stress = 0.16), demonstrating that contaminant signatures can be a useful ecological tool. *I*. *oxyrinchus* are represented with a dark grey triangles, *A*. *vulpinus* with medium grey squares and *P*. *glauca* with a light grey circles. Shark illustration credit: P. Dimens.

Categorical random forest analysis showed that species were distinguishable based on their contaminant signature and were correctly assigned 96% of the time (Table 2). Contaminants that contributed to species separation can be found in the Supplemental Table 1. nMDS showed clear separation among the three species in their contaminant signatures (Fig. 2; stress = 0.16).

**Table 2:** Confusion Matrices for Random Forest Analysis using 10,000 trees with each column demonstrating the number of individuals identified as *P*. *glauca*, *I*. *oxyrinchus* or *A*. *vulpinus* by the analysis.

	*P*. *glauca*	*I*. *oxyrinchus*	*A*. *vulpinus*	Class Error
*P*. *glauca*	21	0	1	0.0454
*I*. *oxyrinchus*	0	45	1	0.0217
*A*. *vulpinus*	2	1	50	0.0566

Out of Bag (OOB) estimate of error rate was 4.13%. Out of bag error estimate is the rate at which the analysis incorrectly identified species.

## Discussion

Several ecological tools are available for studying habitat use and sympatric species resource partitioning. The most appropriate tool depends on the question of interest, the nature of the ecological community, and the available resources. Exploiting the fact that an animal accrues different chemicals signatures based on its distribution and foraging ecology allows us to use chemical tools to determine resource partitioning in seemingly overlapping species and subsequently make inferences on species’ ecological niches. Accumulation of contaminants by predators has consequences for the transport of these chemicals in the environment and the health of their populations. In turn, contaminant accumulation is a product of the interaction of species’ niche, life history, and physiological characteristics. Here, we highlight the ability to use contaminants as an ecological tool to assess niche partitioning and in an accompanying study^33^, we further examine how ecological and physiological factors synergistically influence contaminant accumulation trajectories in three species.

Despite spatial and temporal overlap of *I*. *oxyrinchus*, *P*. *glauca*, *and A*. *vulpinus*^16,45^ in the CCMLE, we were able to discriminate among these three species with high accuracy using both total contaminant loads and profiles. This study corroborates prior findings showing significant differences in ecological niches among these three species in the same area^16^. Preti *et al*.^16^ found differences in stomach contents with *A*. *vulpinus* relying mainly on small epipelagic fish, *P*. *glauca* on squid and other organisms associated with the deep scattering layer and *I*. *oxyrinchus* being intermediate to *A*. *vulpinus* and *P*. *glauca*. Therefore, contaminants can be used as an ecological tool to study niche partitioning in species with varying degrees of spatial, temporal or dietary overlap^18,19,46^.

The strong southern California contaminant signal in all three species (*i*.*e*. DDX proportion) indicates the relative importance of the CCLME to these species’ forage bases and the persistence of DDX in liver tissue. *A*. *vulpinus* are considered to be more coastally oriented^32,47^ than either *P*. *glauca* or *I*. *oxyrinchus*. Therefore, it was not surprising that *A*. *vulpinus* had greater contaminant contributions from DDXs than both *P*. *glauca* and *I*. *oxyrinchus*. Note that details on ontogenetic-related changes in DDX signatures can be found in Lyons *et al*.^33^. A similar patterns relating to DDXs with respect to California coastal residency was found in pinnipeds sampled from southern California^48^, with more coastally associated species having higher DDX proportions and more pelagic species having lower DDX proportions. In comparison, *P*. *glauca* and *I*. *oxyrinchus* are known to spend protracted periods offshore in open waters^12^. However, despite the time spent offshore, DDXs still contributed to a large proportion of their total contaminant burden (>60% on average), suggesting that prey items with strong southern California influences are prominent in the diets of *I*. *oxyrinchus*, *P*. *glauca*, *and A*. *vulpinus*. By contrast, DDX signatures in *Lamna ditropis* (Salmon Sharks), another eastern North Pacific shark species that also utilizes offshore waters and occasionally is found in the CCLME, were much lower than that in *P*. *glauca* or *I*. *oxyrinchus*, with DDXs contributing to only 49% of the total load^34^.

Not only does geographic distribution and diet influence contaminant signatures, but these factors will also influence total contaminant concentrations, which also were distinguishable among species. There was a clear hierarchy in contaminant concentrations among the three species, with *I*. *oxyrinchus* having the highest, followed by *A*. *vulpinus* and then *P*. *glauca*. It is well known that the trophic positions of prey plays a significant role in predator concentrations, with higher trophic level predators having higher accumulated concentrations^49,50^. Of the three species, *I*. *oxyrinchus has* the highest trophic level^16^. Considering the relationship between contaminant concentrations and trophic positioning^49,51^, we confirm previous findings that contaminant concentration can be used to make inferences about ecology.

In addition to foraging ecology and distribution, physiological factors are also expected to influence contaminant accumulation^52,53^; these can, at times, be less emphasized compared to trophic position or trophic linkages in influencing contaminant concentrations among organisms^54,55^. The physiological influence on contaminant concentration is particularly exemplified in the separation between *I*. *oxyrinchus* and *P*. *glauca*, despite overlap in their diets. Although stable isotope analysis has confirmed that juvenile *P*. *glauca* forage at a lower trophic level than juvenile *I*. *oxyrinchus*^56^, adults of both species have been documented to feed on marine mammals^16^. Therefore, while diets overlap, the magnitudes greater contaminant concentrations in *I*. *oxyrinchus*, suggests that other, physiological factors also have an influence on contaminant accumulation. For example, metabolic rates are higher in regionally endothermic *I*. *oxyrinchus* than in ectothermic *P*. *glauca*^57^, which will likely influence contaminant accumulation. In contrast, while *I*. *oxyrinchus* and *A*. *vulpinus* are more physiologically similar (both are regional endotherms and utilize similar matrotrophic reproductive strategies), the discrepancy between them are more likely attributed to trophic differences^16^. Thus, while it is apparent that diet and habitat will influence contaminant signatures and concentrations, to fully explain the differences among species we need to also consider inherent differences in their physiology.

Corroborating the findings of traditional methods, this paper demonstrates that organochlorine contaminants can be used as an additional tool to study niche partitioning in ecological studies^18–20^. Since contaminants are acquired through diet, factors that influence feeding are hypothesized to play an important role in accumulation, and thus niche discrimination. While physiological characteristics dictate energetic demands, ecological characteristics influence where and upon what animals feed. Therefore, contaminant analyses are a tool whereby both ecological and physiological characteristics of a species are incorporated. Recognizing that species may undergo ontogenetic shifts in their ecology or physiology, it is noteworthy that despite the fact that we pooled individuals by species regardless of their sex or age class, we were still able to distinctly discriminate among species. However, details on how contaminant accumulation varied among these species across ontogeny can be found in Lyons *et al*.^33^. Nevertheless, contaminants as ecological markers may serve as a useful tool to provide insight into differential resource usage and physiology within species as well as among them.

## Supplementary information

Supplementary Information

## References

[1] Case TJ, Gilpin ME (1974). Interference Competition and Niche Theory. Proc. Natl. Acad. Sci..

[2] Pianka ER (1981). Competition and niche theory. Ariel.

[3] Schoener TW (1983). Field Experiments on Interspecific Competition. Am. Nat..

[4] Hardin G (1960). The competitive exclusion principle. Science (80-)..

[5] Tilman D (1994). Competition and Biodiversity in Spatially Structured Habitats. Ecology.

[6] Amarasekare P (2003). Competitive coexistence in spatially structured environments: a synthesis. Ecol. Lett..

[7] Pianka ER (1974). Niche overlap and diffuse competition. Proc. Natl. Acad. Sci..

[8] Hurlbert SH (1978). The Measurement of Niche Overlap and Some Relatives. Ecology.

[9] Elbroch LM, Lendrum PE, Newby J, Quigley H, Thompson DJ (2015). Recolonizing wolves influence the realized niche of resident cougars. Zool. Stud..

[10] Matich P (2017). Ecological niche partitioning within a large predator guild in a nutrient-limited estuary. Limnol. Oceanogr..

[11] Downing AS, van Nes EH, Mooij WM, Scheffer M (2012). The Resilience and Resistance of an Ecosystem to a Collapse of Diversity. PLoS One.

[12] Block BA (2011). Tracking apex marine predator movements in a dynamic ocean. Nature.

[13] Oh BZL (2017). Contrasting patterns of residency and space use of coastal sharks within a communal shark nursery. Mar. Freshw. Res..

[14] Winemiller KO (1989). Ontogenetic diet shifts and resource partitioning among piscivorous fishes in the Venezuelan ilanos. Environ. Biol. Fishes.

[15] Papastamatiou YP, Wetherbee BM, Lowe CG, Crow GL (2006). Distribution and diet of four species of carcharhinid shark in the Hawaiian Islands. Mar. Ecol. Prog. Ser..

[16] Preti A (2012). Comparative feeding ecology of shortfin mako, blue and thresher sharks in the California Current. Environ. Biol. Fishes.

[17] Kinney MJ, Hussey NE, Fisk AT, Tobin AJ, Simpfendorfer CA (2011). Communal or competitive? Stable isotope analysis provides evidence of resource partitioning within a communal shark nursery. Mar. Ecol. Prog. Ser..

[18] Ramos R, González-Solís J (2012). Trace me if you can: the use of intrinsic biogeochemical markers in marine top predators. Front. Ecol. Environ..

[19] Chouvelon T (2017). Chemical contaminants (trace metals, persistent organic pollutants) in albacore tuna from western Indian and south-eastern Atlantic Oceans: Trophic influence and potential as tracers of populations. Sci. Total Environ..

[20] Elfes CT (2010). Geographic variation of persistent organic pollutant levels in humpback whale (*Megaptera novaeangliae*) feeding areas of the North Pacific and North Atlantic. Environ. Toxicol. Chem..

[21] Hansen LJ (2004). Geographic variation in polychorinated biphenyl and organochlorine pesticide concentrations in the blubber of bottlenose dolphins from the US Atlantic coast. Sci. Total Environ..

[22] Beaudry MC (2015). Comparative organochlorine accumulation in two ecologically similar shark species (*Carcharodon carcharias* and *Carcharhinus obscurus*) with divergent uptake based on different life history. Environ. Toxicol. Chem..

[23] Braune BM (2005). Persistent organic pollutants and mercury in marine biota of the Canadian Arctic: An overview of spatial and temporal trends. Sci. Total Environ..

[24] Jamieson AJ, Malkocs T, Piertney SB, Fujii T, Zhang Z (2017). Bioaccumulation of persistent organic pollutants in the deepest ocean fauna. Nat. Ecol. Evol..

[25] Jepson PD (2016). PCB pollution continues to impact populations of orcas and other dolphins in European waters. Sci. Rep..

[26] Aznar-Alemany Ò, Giménez J, de Stephanis R, Eljarrat E, Barceló D (2017). Insecticide pyrethroids in liver of striped dolphin from the Mediterranean Sea. Environ. Pollut..

[27] Barón E (2015). Bioaccumulation and biomagnification of classical flame retardants, related halogenated natural compounds and alternative flame retardants in three delphinids from Southern European waters. Environ. Pollut..

[28] Hickie BE, Mackay D, De Koning J (1999). Lifetime pharmacokinetic model for hydrophobic contaminants in marine mammals. Environ. Toxicol. Chem..

[29] Krahn MM (2007). Use of chemical tracers in assessing the diet and foraging regions of eastern North Pacific killer whales. Mar. Environ. Res..

[30] Zeng EY, Venkatesan MI (1999). Dispersion of sediment DDTs in the coastal ocean off southern California. Sci. Total Environ..

[31] Runcie R (2016). A fishery-independent survey of juvenile shortfin mako (*Isurus oxyrinchus*) and blue (*Prionace glauca*) sharks in the Southern California Bight, 1994–2013. Fish. Res..

[32] Cartamil D, Wegner NC, Kacev D, Ben-Aderet N, Kohin S (2010). Movement patterns and nursery habitat of juvenile thresher sharks *Alopias vulpinus* in the Southern California Bight. Mar. Ecol. Prog. Ser..

[33] Lyons Kady, Kacev Dovi, Preti Antonella, Gillett David, Dewar Heidi, Kohin Suzanne (2019). Species-Specific Characteristics Influence Contaminant Accumulation Trajectories and Signatures Across Ontogeny in Three Pelagic Shark Species. Environmental Science & Technology.

[34] Lyons K (2013). Effects of trophic ecology and habitat use on maternal transfer of contaminants in four species of young of the year lamniform sharks. Mar. Environ. Res..

[35] Lyons K (2015). Insights into the life history and ecology of a large shortfin mako shark *Isurus oxyrinchus* captured in southern California. J. Fish Biol..

[36] Lyons K, Lowe CG (2013). Mechanisms of maternal transfer of organochlorine contaminants and mercury in the common thresher shark (*Alopias vulpinus*). Can. J. Fish. Aquat. Sci..

[37] Lyons K, Lowe CG (2013). Quantification of maternal offloading of organic contaminants in elasmobranchs using the histotrophic round stingray (*Urobatis halleri*) as a model. Environ. Sci. Technol..

[38] Reijnders PJH (1994). Toxicokinetics of chlorobiphenyls and associated physiological responses in marine mammals, with particular reference to their potential for ecotoxicological risk assessment. Sci. Total Environ..

[39] Kannan K, Blankenship AL, Jones PD, Giesy JP (2000). Toxicity Reference Values for the Toxic Effects of Polychlorinated Biphenyls to Aquatic Mammals. Hum. Ecol. Risk Assess. An Int. J..

[40] Reddy ML, Reif JS, Bachand A, Ridgway SH (2001). Opportunities for using Navy marine mammals to explore associations between organochlorine contaminants and unfavorable effects on reproduction. Sci. Total Environ..

[41] Schwacke LH (2002). Probabilistic risk assessment of reproductive effects of polychlorinated biphenyls on bottlenose dolphins (Tursiops truncatus) from the Southeast United States Coast. Environ. Toxicol. Chem..

[42] Bursian SJ, Kern J, Remington RE, Link JE, Fitzgerald SD (2013). Dietary exposure of mink (Mustela vison) to fish from the upper Hudson River, New York, USA: Effects on reproduction and offspring growth and mortality. Environ. Toxicol. Chem..

[43] Lyons K, Wynne-Edwards KE (2018). Legacy PCB contamination impairs male embryonic development in an elasmobranch with matrotrophic histotrophy, the Round Stingray (Urobatis halleri). Environ. Toxicol. Chem..

[44] Lyons K, Wynne-Edwards KE (2019). Legacy environmental polychlorinated biphenyl contamination attenuates the acute stress response in a cartilaginous fish, the Round Stingray. Stress.

[45] Holts DB, Julian A, Sosa-Nishizaki O, Bartoo NW (1998). Pelagic shark fisheries along the west coast of the United States and Baja California, Mexico. Fish. Res..

[46] Méndez-Fernandez P (2014). An assessment of contaminant concentrations in toothed whale species of the NW Iberian Peninsula: Part II. Trace element concentrations. Sci. Total Environ..

[47] Cartamil D (2010). Diel movement patterns and habitat preferences of the common thresher shark (*Alopias vulpinus*) in the Southern California Bight. Mar. Freshw. Res..

[48] Blasius ME, Goodmanlowe GD (2008). Contaminants still high in top-level carnivores in the Southern California Bight: Levels of DDT and PCBs in resident and transient pinnipeds. Mar. Pollut. Bull..

[49] Jarman WM, Hobson KA, Sydeman WJ, Bacon CE, McLaren EB (1996). Influence of trophic position and feeding location on contaminant levels in the Gulf of the Farallones food web revealed by stable isotope analysis. Environ. Sci. Technol..

[50] Fisk AT, Tittlemier SA, Pranschke JL, Norstrom RJ (2002). Using anthropogenic contaminants and stable isotopes to assess the feeding ecology of Greenland Sharks. Ecology.

[51] Hop H, Borgå K, Gabrielsen GW, Kleivane L, Skaare JU (2002). Food Web Magnification of Persistent Organic Pollutants in Poikilotherms and Homeotherms from the Barents Sea. Environ. Sci. Technol..

[52] Borgå K (2012). Trophic magnification factors: Considerations of ecology, ecosystems, and study design. Integr. Environ. Assess. Manag..

[53] Nichols JW (1993). Physiologically-based toxicokinetic modeling of three waterborne chloroethanes in channel catfish, Ictalurus punctatus. Aquat. Toxicol..

[54] Vander Zanden MJ, Rasmussen JB (1996). A Trophic Position Model of Pelagic Food Webs: Impact on Contaminant Bioaccumulation in Lake Trout. Ecol. Monogr..

[55] Koelmans AA, Van der Heijde A, Knijff LM, Aalderink RH (2001). Integrated Modelling of Eutrophication and Organic Contaminant Fate & Effects in Aquatic Ecosystems. A Review. Water Res..

[56] Madigan DJ (2012). Stable isotope analysis challenges wasp-waist food web assumptions in an upwelling pelagic ecosystem. Sci. Rep..

[57] Bernal D (2003). Comparative studies of high performance swimming in sharks II. Metabolic biochemistry of locomotor and myocardial muscle in endothermic and ectothermic sharks. J. Exp. Biol..

