# Supplementary material for: Organic contaminants as an ecological tool to explore niche partitioning: a case study using three pelagic shark species
Source: Sci Rep. 2019 Aug 19;9:12080. doi: 10.1038/s41598-019-48521-6 (PMC6700177; doi:10.1038/s41598-019-48521-6)
Supplement: Supplementary file 1 — Supplementary Information [file 41598_2019_48521_MOESM1_ESM.docx]

**Title:** Organic contaminants as an ecological tool to explore niche partitioning: a case study using three pelagic shark species

**Authors:** Kady Lyons^1*^, Dovi Kacev^2,3^, Antonella Preti^2,4^, David Gillett^3^, Heidi Dewar^2^

^1*^Georgia Aquarium, 225 Baker Street NW, Atlanta, Georgia 30313

[kady.lyons@sbcglobal.net](mailto:kady.lyons@sbcglobal.net)

^2^Southwest Fisheries Science Center, [dovi.kacev@gmail.com](mailto:dovi.kacev@gmail.com), heidi.dewar@noaa.gov

^3^Southern California Coastal Water Research Project, [davidg@sccwrp.org](mailto:davidg@sccwrp.org)

^4^University of California Santa Cruz, [antonella.preti@noaa.gov](mailto:antonella.preti@noaa.gov)

**Supplemental information**

Table 1. List of contaminants ranked by their significance and associated gini value from the random forest analysis. Higher values indicate more importance in terms of separating species based on contaminant signatures.

| Contaminant | Gini Value | p value |
| --- | --- | --- |
| Alpha chlordane | 0.0085 | 0.0099 |
| Beta BHC | 0.0034 | 0.0099 |
| Hexachlorobenzene | 0.0050 | 0.0099 |
| Mirex | 0.0022 | 0.0099 |
| PCB101 | 0.0202 | 0.0099 |
| PCB105 | 0.0083 | 0.0099 |
| PCB110 | 0.0435 | 0.0099 |
| PCB118 | 0.0067 | 0.0099 |
| PCB128 | 0.0136 | 0.0099 |
| PCB141 | 0.0055 | 0.0099 |
| PCB151 | 0.0038 | 0.0099 |
| PCB157 | 0.0067 | 0.0099 |
| PCB158 | 0.0109 | 0.0099 |
| PCB167 | 0.0053 | 0.0099 |
| PCB174 | 0.0075 | 0.0099 |
| PCB177 | 0.0042 | 0.0099 |
| PCB187 | 0.0058 | 0.0099 |
| PCB194 | 0.0060 | 0.0099 |
| PCB52 | 0.0086 | 0.0099 |
| PCB66 | 0.0064 | 0.0099 |
| PCB70 | 0.0043 | 0.0099 |
| PCB87 | 0.0127 | 0.0099 |
| PCB95 | 0.0187 | 0.0099 |
| PCB97 | 0.0074 | 0.0099 |
| PCB99 | 0.0125 | 0.0099 |
| Total DDT | 0.0320 | 0.0099 |
| Total PCB | 0.0495 | 0.0099 |
| Total Pesticides | 0.0415 | 0.0099 |
| 2,4’-DDE | 0.0281 | 0.0099 |
| 2,4’-DDT | 0.0085 | 0.0099 |
| 4,4’-DDE | 0.0081 | 0.0099 |
| Gamma Chlordane | 0.0016 | 0.0198 |
| PCB149 | 0.0031 | 0.0198 |
| PCB200 | 0.0056 | 0.0198 |
| PCB201 | 0.0026 | 0.0198 |
| 4,4’-DDE | 0.0019 | 0.0198 |
| 4,4’-DDMU | 0.0021 | 0.0198 |
| PCB74 | 0.0026 | 0.0297 |
| Trans-nonachlor | 0.0015 | 0.0297 |
| PCB156 | 0.0027 | 0.0396 |
| PCB189 | 0.0003 | 0.0396 |
| PCB183 | 0.0014 | 0.0495 |
| PCB49 | 0.0015 | 0.0495 |
| PCB123 | 0.0006 | 0.0594 |
| PCB31 | 0.0007 | 0.0594 |
| 4,4’-DDT | 0.0006 | 0.0594 |
| Cis-nonachlor | 0.0009 | 0.0693 |
| PCB126 | 0.0005 | 0.0693 |
| PCB170 | 0.0009 | 0.0693 |
| PCB168&132 | 0.0004 | 0.0792 |
| PCB44 | 0.0002 | 0.0792 |
| PCB56 | 0.0001 | 0.0792 |
| PCB195 | 0.0004 | 0.1089 |
| PCB28 | 0.0005 | 0.1188 |
| 2,4’-DDD | 0.0002 | 0.1584 |
| PCB180 | 0.0002 | 0.1683 |
| PCB114 | 0.0001 | 0.1782 |
| PCB206 | 0.0001 | 0.1782 |
| PCB209 | 0.0001 | 0.1782 |
| PCB138 | 0.0004 | 0.1980 |
| PCB33 | 0.0000 | 0.2475 |
| Oxychlordane | 0.0000 | 0.5743 |
| PCB77 | 0.0000 | 1.0000 |
| Endrin Aldehyde | 0.0000 | 1.0000 |
